# Laparoscopic Appendectomy in Children: Experience in a Single Centre in Chittagong, Bangladesh

**DOI:** 10.1155/2014/125174

**Published:** 2014-03-11

**Authors:** Md. Jafrul Hannan

**Affiliations:** Department of Pediatric Surgery, South Point Hospital, 11 Access Road, Chotopul, Agrabad, Chittagong 4100, Bangladesh

## Abstract

*Background*. Since the latter half of 1980s laparoscopy has become a well accepted modality in children in many surgical procedures including appendectomy. We present here the experience of laparoscopic appendectomy in children in a tertiary care hospital in Bangladesh.  *Subjects & Methods*. From October 7, 2005 to July 31, 2012, 1809 laparoscopic appendectomies were performed. Laparoscopy was performed in all the cases using 3 ports. For difficult and adherent cases submucosal appendectomy was performed. Feeding was allowed 6 h after surgery and the majority was discharged on the first postoperative day. The age, sex, operative techniques, operative findings, operative time, hospital stay, outcome, and complications were evaluated in this retrospective study. *Results*. Mean age was 8.17 ± 3.28 years and 69% were males. Fifteen percent were complicated appendicitis, 8 cases needed conversion, and 27 cases were done by submucosal technique. Mean operating time was 39.8 ± 14.2 minutes and mean postoperative hospital stay was 1.91 days. About 5% cases had postoperative complications including 4 intra-abdominal abscesses. *Conclusions*. Laparoscopic appendectomy is a safe procedure in children even in complicated cases.

## 1. Introduction

Semm, a German Gynecologist, first described the method of laparoscopic appendectomy in adults [1]. Since then a series of reports came out with restrained enthusiasm for laparoscopy [2–6]. In children, the doubts were more pronounced especially in complicated cases [7–11]. However practice of laparoscopy increased with passage of time and its superiority over open technique is now well established in terms of morbidity, recovery, wound infections, hospital stay, and utility in young females and obese children [6, 12–15]. Nowadays single incision laparoscopy is also being practiced [13]. We describe here our experience with laparoscopic appendectomy in children of Bangladesh using conventional 3 port techniques.

## 2. Subjects and Methods

From October 7, 2005 to July 31, 2012, 1809 laparoscopic appendectomies were performed.

**Figure 1:**
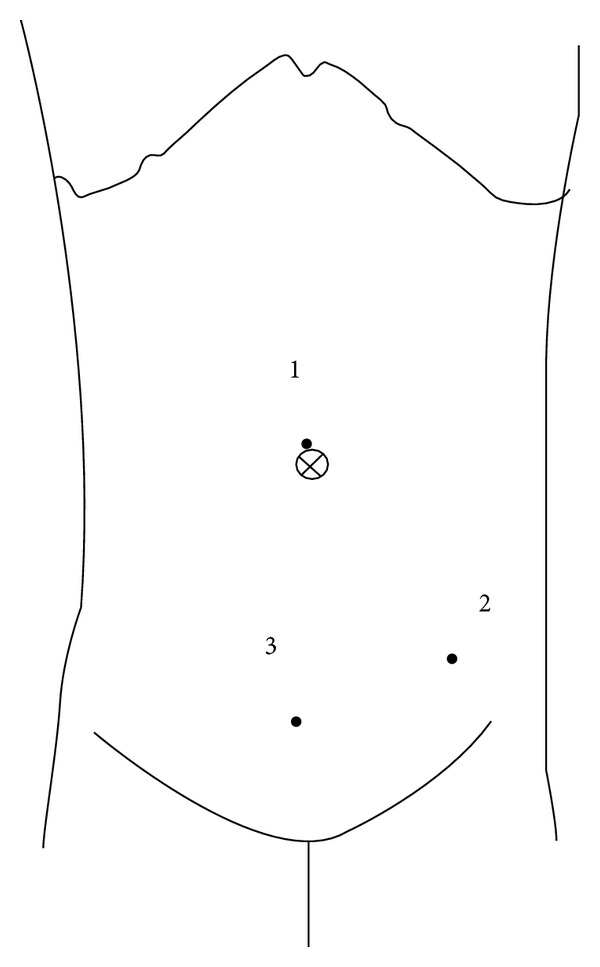
Ports placement.

Diagnoses were based on clinical suspicion as well as on ultrasonogram findings. Under general endotracheal anesthesia, laparoscopy was performed in all the cases. Patients were supine with monitor on the right side and surgeon on the left side of midsection of patient's body. Assistant stood on the right side of surgeon towards head-end of patient. Three ports were placed: supraumbilical port for telescope, one port just medial to and below the left anterior superior iliac spine, and another just above and to the right of pubic crest (Figure 1). The telescope was 5 mm 30° in children below 5 years and 10 mm 30° for those above 5 years. The supraumbilical port was introduced by open technique and insufflations were done by keeping CO_2_ pressure between 10 and 15 mmHg. After port placement and insufflations, the right side and foot end of the patient was elevated. For high-up and subhepatic appendix, head-end of the patient needed to be elevated and, on occasions, a fourth port was needed in left flank for retraction of intestines. Bipolar cautery was used to burn the mesoappendix before skeletonization, using monopolar hook cautery. For perforated appendix, purulent peritoneal fluid was collected for culture and sensitivity before proceeding to appendectomy. For difficult and adherent cases where no plane could be established between appendices and surrounding structures, submucosal appendectomy was performed [16]. Intracorporeal knotting with 2/0 or 3/0 vicryl was used to ligate the base of appendix before division and retrieval. Appendix was retrieved in a cut glove finger to avoid contamination in perforated cases. After peritoneal lavage in perforated cases and in those with submucosal appendectomy, a PVC 14F size drain was kept before port closure. The drain was removed 48 to 72 hours postoperatively in perforated cases and after 24 hours in submucosal appendectomy cases where there was no perforation. Ports were closed using the same thread subcuticularly after fascial closure at supraumbilical port. Feeding was allowed 6 hours after surgery, and the majority of the patients were discharged on the first postoperative day. Followups were at 1 week, 1 month, 3 months, 6 months, and 1 year.

The age, sex, operative techniques, operative findings, operative time, hospital stay, outcome, and complications were evaluated. The Ethical Review Committee for Thesis and Research of Chattagram Maa-O-Shishu Hospital Medical College gave permission to conduct this retrospective study.

## 3. Results

Ages of the patients ranged from 6 months to 16 years (mean 8.17 ± 3.28 years), 70% were between 5 and 10 years, and 1066 (69%) were males. Out of 1809 cases 273 (15.1%) were complicated appendicitis. Twenty-seven cases had extra-appendicular pathologies (Table 1). Twenty-seven cases were done by submucosal technique and eight needed conversion to open technique. Mean operating time was 39.8 ± 14.2 minutes (range 20 to 90 minutes). Overall 5.04% cases had some complications including 18 postoperative ileus, 20 port-site infections (PSI), and 4 intra-abdominal abscesses (IAA). During follow-up period, 49 cases came with complaints of abdominal pain of which 31 were diagnosed as urinary tract infection; 2 cases had ovarian cysts and remainder with nonspecific abdominal pain. Mean postoperative hospital stay was 1.91 days.

## 4. Discussion

Since October 2005 laparoscopy is the primary modality in our centre for the treatment of appendicitis. During the study period 308 children were operated by open method, principally due to nonavailability of laparoscope and also in some cases due to parental refusal. For uncomplicated cases we did not find significant differences in operating time and hospital stay between our study population and open cases. Although there are reports mostly from the early laparoscopic era that laparoscopy requires longer operating time, recent studies prove the opposite [13, 14, 17–21]. Operating time for our laparoscopically performed complicated cases was less than those done by open method. The conversion rate in our series was kept to a minimum by adopting a new technique in difficult situations, that is, submucosal appendectomy [16]. Our policy with appendix mass was to proceed with primary intervention using laparoscopy and we did not face much difficulty during operation [22].

Postoperative hospital stay in our series was on average less than 2 days. In the majority of uncomplicated appendicitis, patients were discharged on the following day after operation. Longer stay up to 5 days was required in 4 cases that developed postoperative ileus or wound Infection [23]. However complicated appendicitis particularly those with perforation and peritonitis were kept at least for 3 days postoperatively for parenteral antibiotics [16]. Fourteen with ileus and some with wound infection and intra-abdominal abscesses needed longer to stay up to 20 days (mean 5.55 ± 1.72 days).

Extra-appendicular pathologies found during laparoscopy were managed accordingly (Table 1). Ages of 3 intussusceptions cases were 4, 5, and 7 years and no lead point could be found. These children were doing well during the study period. The findings of scattered tubercles all over the peritoneal cavity including on intestinal surfaces and inside the abdominal wall prompted us to the diagnosis of tuberculosis. Tubercles from abdominal wall and from mesentery were sent for histopathology and appendectomy was not performed. Appendectomy was performed in 24 of 27 extra-appendicular cases and histopathology revealed appendicitis in one case that was one of mesenteric lymphadenitis cases. Diagnosis of appendicitis in our centre was primarily based on clinical impression. Ultrasonogram reports were available in 875 cases including 17 with extra-appendicular pathology. We did not send for histopathology of perforated and grossly inflamed appendices. Records of histopathology of 335 uncomplicated appendicitis cases showed 23 (6.86%) normal appendices.

While overall infection rate in our study group including PSI and IAA was 2.54%, it was 7.32% in complicated cases. However, both are lower than in the open cases done during this period (7.46% and 18.86%, resp.). The overall infection rate and infection in complicated cases are significantly less in laparoscopy group (*P* < 0.001 and *P* < 0.006, resp.). Careful attention to avoid port-site contamination in perforated cases including use of cut glove finger during retrieval was a contributing factor in this regard. Peritoneal toileting in perforated cases was thorough and did not pose any difficulty in our cases which might be a reason behind small number of IAA. We have managed two IAA cases conservatively and two by per-rectal drainage [24]. Although there is a consensus about use of laparoscopy in complicated cases, few recent studies showed increased chance of IAA [14, 25–29]. None of our uncomplicated cases developed IAA. Just over 5% complication rate in our series is quite acceptable, while it included conversion and abdominal pain during followup [20]. We did not find any particular reason behind occurrence of urinary tract infections in the study group during follow-up period. However 22 of 31 cases with urinary tract infection were girls. The nonspecific abdominal pain was diagnosed when patients came with abdominal pain and no specific pathology was found on ultrasonogram and clinically.

Limitation in this study was its lack of a proper group with open appendectomy to compare the results more authentically.

## 5. Conclusions

Laparoscopic appendectomy is a safe procedure in children even in complicated cases.

## Figures and Tables

**Table 1:** Extra-appendicular pathologies found at laparoscopy^†^.

Findings	Number	Management
Mesenteric lymphadenitis	07	Lymph node biopsy*
Worm bolus	04	Squeezed into colon
Intussusceptions	03	Reduced
Tuberculosis	03	Tubercles taken for biopsy
Mesenteric gap with herniation	02	Repaired
Omental infarction	01	Excised
Twisted ovarian cyst	03	Untwisted and cystectomy done
Leaking corpus luteal cyst	04	Left as it was
Total	**27**	

^†^Appendectomy was also done in all except tuberculosis cases.

*Two cases were diagnosed as non-Hodgkin's Lymphoma.
